# Genome-centric investigation of bile acid-metabolizing microbiota in chickens and their association with *Eimeria tenella* and *Salmonella typhimurium* infections

**DOI:** 10.3389/fvets.2025.1669620

**Published:** 2025-10-17

**Authors:** Kai-Meng Shang, Hany M. Elsheikha, Yong-Jie Wei, Xiao-Xuan Zhang, Xin-Wen Hou, Hai-Long Yu, Yanan Cai, Hong-Bo Ni, Rui Liu, He Ma, Jing Jiang, Fulong Nan, Xing Yang

**Affiliations:** ^1^Integrated Laboratory of Pathogenic Biology, College of Preclinical Medicine, Dali University, Dali, China; ^2^College of Life Sciences, Changchun Sci-Tech University, Shuangyang, Jilin Province, China; ^3^College of Veterinary Medicine, Qingdao Agricultural University, Qingdao, Shandong, China; ^4^Faculty of Medicine and Health Sciences, School of Veterinary Medicine and Science, University of Nottingham, Loughborough, United Kingdom; ^5^College of Veterinary Medicine, Jilin Agricultural University, Changchun, Jilin, China

**Keywords:** chicken, bile acid metabolism, *Salmonella typhimurium*, *Eimeria tenella*, gut microbiota

## Abstract

**Introduction:**

Bile acid (BA) metabolism by gut microbiota plays a crucial role in host health by influencing nutrient absorption, immune responses, and resistance to pathogens. Elucidating how enteric infections disrupt the BA-microbiota axis is crucial for advancing microbiota-based therapeutics, precision nutrition, and post-antibiotic disease control strategies.

**Methods:**

We reconstructed 9,990 high-quality microbial genomes from the gut microbiota of chicken and performed genome-resolved metabolic profiling. Comparative analyses were conducted across host species, including humans and pigs. Also, 135 intestinal samples collected from different regions of the chicken gut were analyzed. Additional samples from chickens infected with Salmonella typhimurium and Eimeria tenella were included to assess infection-associated alterations.

**Results:**

Our results reveal that the phylum *Bacillota_A* is predominant, with key BA-transforming enzymes, including bile salt hydrolase (BSH) and 7α-hydroxysteroid dehydrogenase (7α-HSDH), present in a substantial proportion of the genomes. Chickens harbored a higher proportion of BSH genes compared to humans and pigs, with *Ligilactobacillus* and *Alistipes* identified as major contributors. Region-specific analysis showed that BA-metabolizing microbes are unevenly distributed along the intestinal tract, with the highest diversity observed in the cecum and colon. Experimental pathogen challenges revealed that *S. typhimurium* infection altered BSH gene abundance and overall microbial community structure, whereas *E. tenella* infection increased taxonomic richness but reduced community evenness.

**Discussion:**

Together, these findings advance our understanding of microbial contributions to BA dynamics in poultry and offer insights into the role of BA metabolism in gut health and pathogen resistance.

## 1 Introduction

Bile acids (BAs) are key regulators of gastrointestinal physiology. Synthesized from cholesterol in the liver and secreted into the small intestine, they facilitate lipid digestion, nutrient absorption and metabolic signaling (1, 2). Beyond these host-mediated roles, BAs also act as potent ecological agents in the gut, exerting antimicrobial pressure that shapes microbial community structure and function (3–5). In response, gut microbes have evolved enzymatic strategies to transform BAs, beginning with bile salt hydrolase (*BSH*)-mediated deconjugation and extending to more complex conversions such as oxidation and 7α/*β*-dehydroxylation. These transformations are catalyzed by enzymes including 7α-hydroxysteroid dehydrogenase (*7α-HSDH*) and bile acid-CoA ligase (*baiB*) (3, 4, 6).

These microbial transformations alter BA structure, toxicity, and receptor-mediated signaling, thereby influencing host physiology, immune responses, pathogen resistance and metabolic health (7–9). In mammals, particularly humans and pigs, BA transformation pathways are increasingly recognized as central to gut homeostasis, with implications for conditions such as inflammatory bowel disease and metabolic syndrome (10, 67). In contrast, despite the economic and scientific importance of poultry and growing interest in their gut microbiota (11–15), the mechanistic understanding of BA–microbiota interactions in birds remains limited. This disparity highlights a critical knowledge gap in avian gut biology.

The chicken (*Gallus gallus domesticus*) serves as both a cornerstone of global protein production and a valuable model for gut microbiome research. Its distinctive gastrointestinal anatomy, including rapid digesta transit (16) and paired ceca, create a unique ecological niche for microbial colonization and metabolic specialization. However, the diversity, distribution, and functional capacity of BA-transforming microbes in chickens, particularly at the genome-resolved level, remain poorly characterized. This limits efforts to harness the gut microbiota for improved nutrient utilization, growth performance and disease resistance. Interestingly, BA metabolism plays a crucial role in shaping poultry health (17), with BAs modulating both innate and adaptive immune responses via interaction with immune cells and cytokines (18). Disruptions in BA signaling may therefore impair host immunity and increase susceptibility to disease.

Among the most significant health challenges in poultry are salmonellosis and coccidiosis. Salmonellosis remains a leading cause of both acute and chronic systemic infections, resulting in major economic losses to the poultry industry (19, 20). Coccidiosis, traditionally managed using anticoccidial drugs, is becoming harder to control due to rising drug resistance, driving interest in alternatives such as medicinal plants (21–25). Infections caused by enteric pathogens such as *Salmonella typhimurium* and *Eimeria tenella* can disrupt gut microbial communities and alter BA composition and availability, impairing digestion, immune signaling, and colonization resistance (18, 26–28). Understanding how such infections perturb the BA-microbiota axis is critical for developing next-generation interventions, including microbiota-based therapies, precision nutrition and post-antibiotic disease control strategies.

In this study, we present the most comprehensive genome-resolved metagenomic analysis of the chicken gut microbiota to date. By reconstructing and analyzing nearly 10,000 high-quality genomes, we systematically characterized the taxonomic and functional repertoire of BA-metabolizing microorganisms in the chicken intestine. We examined region-specific patterns of BA metabolism along the intestinal tract, compared host-specific BA pathways across chickens, humans, and pigs, and evaluated how common poultry infections, such as *S. typhimurium* and *E. tenella*, affect the BA-transforming potential of the gut microbiome. Our findings offer new insights into the microbial ecology of BA metabolism in chickens and identify key taxa and pathways linked to both health and disease. This work establishes a foundation for microbiome-informed strategies aimed at improving poultry resilience, productivity, and welfare in the context of reduced antibiotic use and rising global food demand.

## 2 Materials and methods

### 2.1 Data collection

We utilized 25,827 microbial genomes previously collected in our laboratory from an in-house microbial genome database (29). Additionally, 135 intestinal samples were obtained from multiple anatomical regions of the chicken gut, including the duodenum, jejunum, ileum, cecum, and colorectum (30, 66). To test the hypothesis that enteric infections can disrupt the gut microbial community and impair BA metabolism, we collected 10 samples from chickens infected by *S. typhimurium* (31) and 8 samples from chickens infected by *E. tenella* (32) (Supplementary Table 1).

### 2.2 Preprocessing and bioinformatic analysis

To ensure high-quality sequencing data, raw reads from the samples underwent quality control using fastp (33) (v0.23.0) with the parameters: -q 20 -u 30 -n 5 -y -Y 30 -l 80 --trim_poly_g. Host-derived sequences were removed by aligning the reads to the chicken reference genome (NCBI RefSeq assembly: GCF_016699485.2) using Bowtie2 (34) (v2.5.0). Clean reads were retained for downstream analyses. The 25,827 genomes, including metagenome-assembled genomes (MAGs) and cultured isolates, were evaluated for completeness and contamination using CheckM2 (35) (v1.0.1). Genomes with ≥80% completeness and ≤5% contamination were classified as high quality. Strain-level dereplication was performed with dRep (36) (v3.4.3) at 99% average nucleotide identity (ANI), using the parameters: -pa 0.9 -sa 0.99 -nc 0.30 -cm larger --S_algorithm fastANI. Taxonomic classification was conducted using the classify_wf workflow in GTDB-Tk (37) (v2.3.2) with the GTDB reference database.

### 2.3 Functional analysis of BA-related microbial genes

Open reading frames (ORFs) were predicted from the dereplicated genomes using Prodigal (38) (v2.6.3). Functional annotation was performed by aligning the predicted protein sequences to the KEGG database using DIAMOND (39) (v2.1.8), selecting the top hit based on the highest bit score. KEGG Orthologs (KOs) involved in secondary BA biosynthesis (KEGG pathway: map00121) were extracted for targeted analysis. Gene copy numbers and their genomic origins were determined from the KO annotations. To quantify gene abundance, high-quality reads (20 million per sample) were mapped to the nonredundant microbial gene catalog using Bowtie2. The read counts were normalized to transcripts per kilobase million (TPM) by accounting for both gene length and sequencing depth following the standard procedure (40, 41).

### 2.4 Statistical analyses and visualization

All statistical analyses were performed in R (v4.2.2). Rarefaction curves were generated using the vegan package (v2.6–4). Diversity indices, including Shannon, Richness, and Simpson, were calculated based on both taxonomic and functional gene abundance data. *β*-diversity was evaluated via Principal Coordinate Analysis (PCoA) using Bray–Curtis distance. Group differences were evaluated using permutational multivariate analysis of variance (PERMANOVA). The Wilcoxon rank-sum test was used to determine significant differences in diversity indices and the relative abundance of taxa and functional genes across groups. *p*-values for pairwise taxonomic comparisons were adjusted for multiple testing using the false discovery rate (FDR) method implemented in R with p.adjust (p, method = “fdr”). Sankey plots were generated with the ggsankey package (v0.0.9), and all other visualizations were produced using ggplot2 (v4.2.3) (42).

## 3 Results

### 3.1 Genomes involved in BA transformation pathways in the chicken intestine

**Figure 1:**
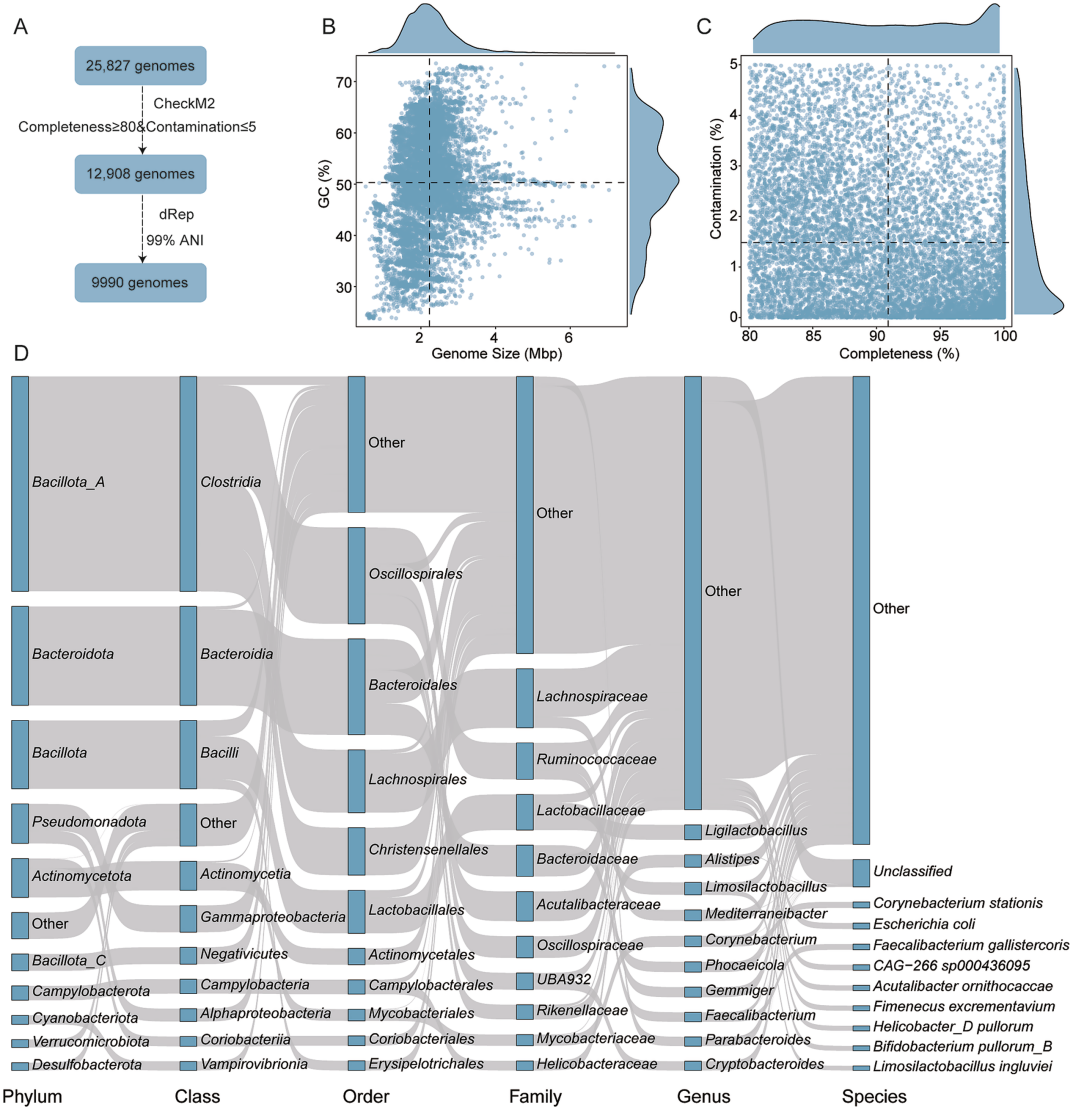
Genomic overview of microbial communities analyzed. **(A)** Workflow illustrating the genome identification, quality control, and dereplication steps. **(B,C)** Genomic characteristics of the final 9,990 high-quality genomes, including genome size, GC content, completeness and contamination. **(D)** Taxonomic composition of genomes predicted to participate in BA transformation pathways. Each rectangle represents a taxonomic rank, with its length proportional to the number of genomes assigned to that category.

To establish a comprehensive genomic profile of the chicken gut microbiota, a total of 25,827 genomes were initially retrieved. After quality filtering (≥80% completeness and ≤5% contamination), 12,908 genomes were retained. Dereplication at a 99% ANI threshold yielded 9,990 non-redundant, high-quality genomes for downstream analysis (Figure 1A). These genomes ranged in size from 0.50 to 7.29 Mbp (average: 2.23 Mbp), with GC content between 23.71 and 73.55% (average: 50.29%) (Figure 1B). Mean completeness was 90.92% and mean contamination was 1.48% (Figure 1C and Supplementary Table 2). Taxonomic classification revealed that these genomes spanned 23 phyla, 192 families, and 708 genera. The most dominant phylum was *Bacillota_A* (39.62%, *n* = 3,958), followed by *Bacteroidota* (18.24%, *n* = 1,822) and *Bacillota* (12.59%, *n* = 1,258). According to the Genome Taxonomy Database (GTDB), *Bacillota* and *Bacillota_A* are distinct but phylogenetically related phylum-level lineages. The “_A” suffix is used by GTDB to denote a separate clade that was split from the original *Bacillota* to preserve monophyly based on genome-wide phylogenetic analysis. At the family level, *Lachnospiraceae* (10.88%, *n* = 1,087), *Ruminococcaceae* (6.71%, *n* = 670), and *Lactobacillaceae* (6.56%, *n* = 655) were most prevalent. The leading genera included *Ligilactobacillus* (2.72%, *n* = 272), *Alistipes* (2.28%, *n* = 228), and *Limosilactobacillus* (2.28%, *n* = 228) (Figure 1D and Supplementary Table 2). The broad range of genome sizes and GC content supports the presence of both fast-growing low-GC organisms and more genetically stable high-GC taxa. This diversity serves as a foundation for the metabolic specialization observed in BA transformation pathways.

### 3.2 Role of genomes in BA metabolism in the chicken intestine

**Figure 2:**
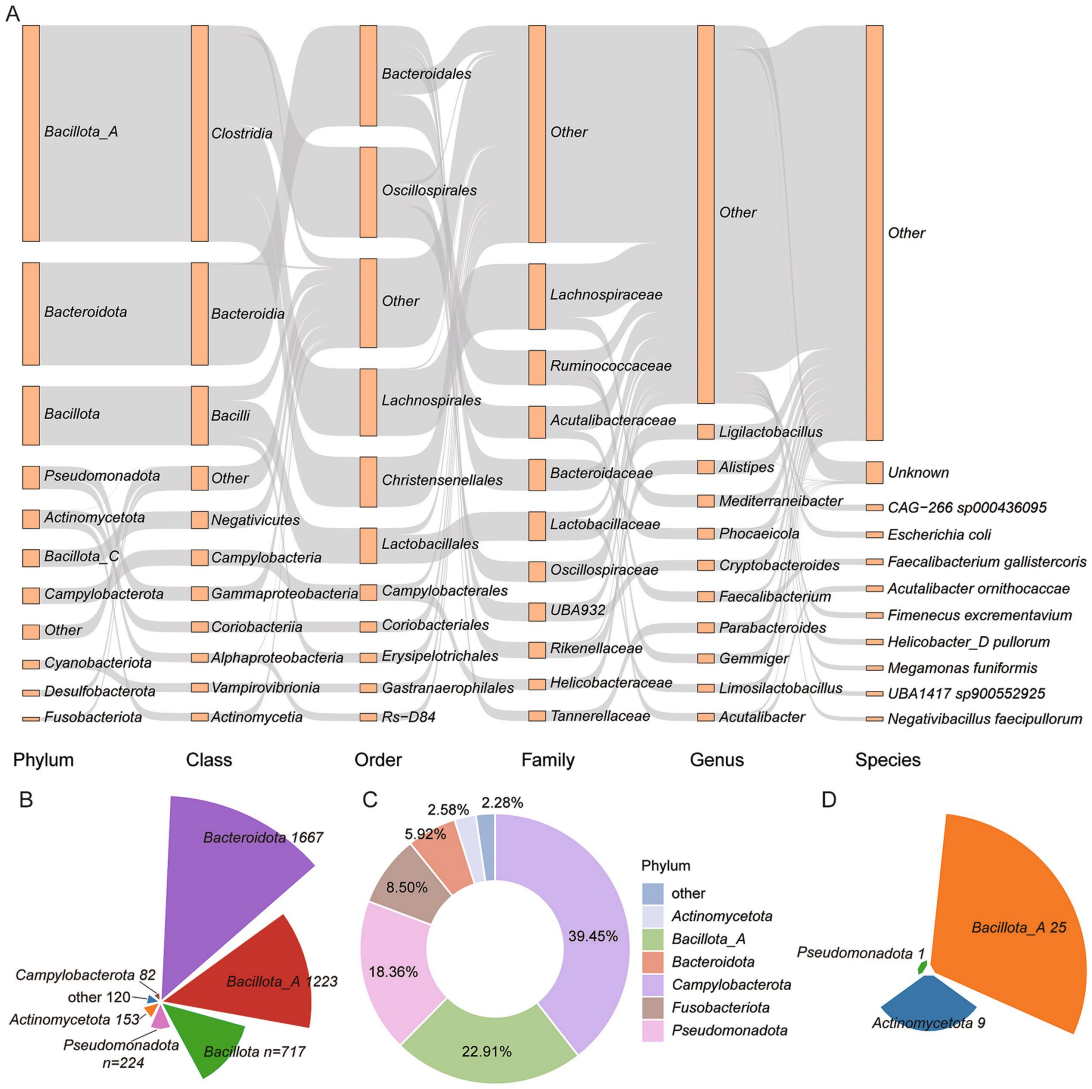
BA transformation potential across 8,009 genomes. **(A)** Taxonomic distribution of genomes carrying BA transformation genes. Taxonomic levels are shown as rectangles, with lengths proportional to the number of genomes assigned to each category. **(B–D)** Proportions of genomes encoding key enzymes involved in BA metabolism: **(B)** Bile Salt Hydrolase (*BSH*), **(C)** 7α-hydroxysteroid dehydrogenase (*7α-HSDH*), and **(D)** bile acid-CoA ligase (*baiB*).

Among the 9,990 genomes, 8,009 (80.17%) were annotated as carrying genes involved in BA transformation pathways, including deconjugation, oxidation and dehydroxylation (Figure 2A and Supplementary Table 3). Specifically, 4,186 genomes encoded *BSH*, the enzyme responsible for bile salt deconjugation. These *BSH*-carrying genomes were distributed across 12 phyla, with *Bacteroidota* (*n* = 1,667), *Bacillota_A* (*n* = 1,223), and *Bacillota* (*n* = 717) being the most abundant (Figure 2B). At the family level, *Lachnospiraceae* (*n* = 575), *Bacteroidaceae* (*n* = 513), and *Lactobacillaceae* (*n* = 463) predominated. Genus-level analysis identified *Ligilactobacillus* (*n* = 238) and *Alistipes* (*n* = 222) as key contributors (Supplementary Table 3). In contrast, fewer genomes encoded enzymes involved in downstream BA transformations. Only nine phyla harbored *7α-HSDH*, which catalyzes hydroxyl oxidation. These included *Campylobacterota* (*n* = 260), *Bacillota_A* (*n* = 151), and *Pseudomonadota* (*n* = 121) (Figure 2C). Additionally, *baiB*, involved in 7α/*β*-dehydroxylation, was detected in only three phyla: *Bacillota_A* (*n* = 25), *Actinomycetota* (*n* = 9), and *Pseudomonadota* (*n* = 1) (Figure 2D). These findings indicate that while deconjugation is widespread across the chicken gut microbiota, the capacity for complete secondary BA modification is restricted to a relatively narrow set of taxa.

### 3.3 Host-specificity of BA-metabolizing microorganisms in chicken

**Figure 3:**
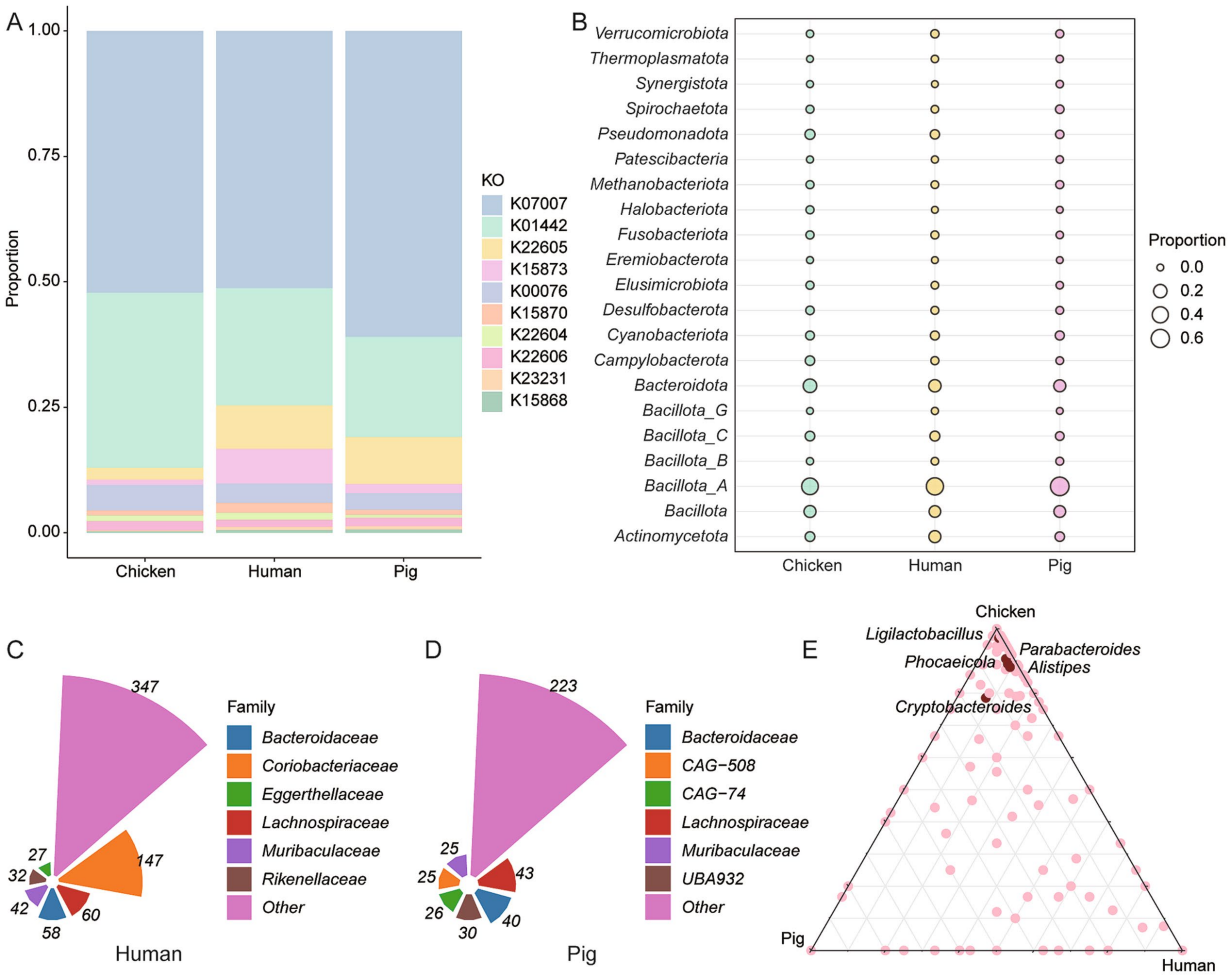
Host-specific BA metabolism by intestinal microbiota. **(A)** Comparative analysis of BA metabolism-related gene abundance in chickens, humans and pigs. **(B)** Phylum-level classification of *BSH*-encoding genomes. **(C,D)** Distribution of BA-metabolizing bacterial families in the human **(C)** and pig **(D)** gut microbiomes. **(E)** Genus-level classification of *BSH*-carrying genomes across the intestinal microbiota of chickens, humans and pigs.

To assess host-specific differences in BA-metabolizing microbiota, we compared the 9,990 high-quality chicken intestinal genomes to publicly available humans [2,294 MAGs; (43)] and pigs [1,411 MAGs, (44)]. Functional annotation revealed 3,499 BA-related KOs in 1,741 human MAGs and 2,229 KOs in 1,162 pig MAGs (Supplementary Table 4). Chickens exhibited the highest proportion of *BSH* gene-related genes but the lowest proportion of *baiA* (K22605) genes (Figure 3A). Across all three hosts, *Bacillota_A* was the dominant BA-metabolizing phylum, comprising 49.63% of human, 63.68% of pig, and 44.64% of chicken genomes (Figure 3B). *BSH* genes were widely distributed, present in 40.95% of human and 35.46% of pig MAGs (Supplementary Table 4). At the family level, *Coriobacteriaceae* was most abundant among BA metabolizers in humans, while *Lachnospiraceae* dominated in pigs (Figures 3C,D). In chickens, *BSH* gene-carrying genera such as *Ligilactobacillus*, *Parabacteroides*, *Phocaeicola*, *Alistipes*, and *Cryptobacteroides* were more prevalent compared to the human and pig datasets (Figure 3E).

### 3.4 Region-specific BA-metabolizing potential along the chicken intestine

**Figure 4:**
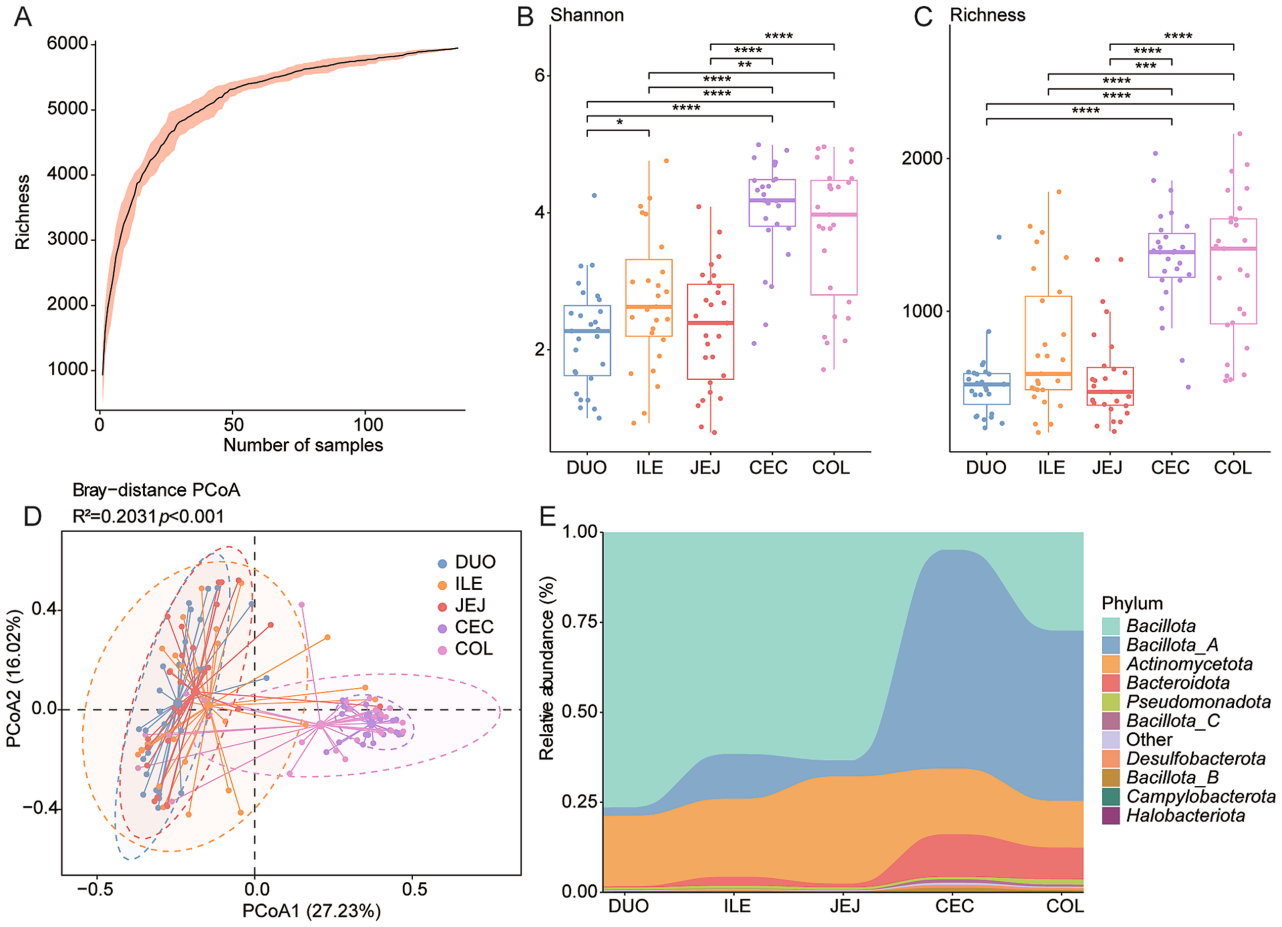
Spatial variation of BA-metabolizing microbiota along the chicken intestine. **(A)** Rarefaction curves illustrating sequencing depth and species richness across different intestinal regions. **(B,C)** Boxplots of Shannon diversity and species of microbial communities in five gut regions. **(D)** Principal Coordinates Analysis (PCoA) based on Bray–Curtis distances showing microbial *β*-diversity by intestinal site. **(E)** Stacked bar plots representing phylum-level taxonomic composition across the duodenum (DUO), jejunum (JEJ), ileum (ILE), cecum (CEC), and colon (COL). Statistical significance was determined using Wilcoxon rank-sum test: *p < 0.05; **p < 0.01; ***p < 0.001.

Microbial diversity and BA-metabolizing potential were analyzed across five intestinal regions: duodenum, jejunum, ileum, cecum, and colon. Rarefaction analysis confirmed sufficient sequencing depth (Figure 4A). Alpha diversity (Shannon and richness indices) revealed significantly lower microbial diversity in the small intestine compared to the cecum and colon (*p* < 0.05, Wilcoxon rank-sum test; Figures 4B,C). PCoA based on Bray–Curtis distances showed distinct microbial community structures across regions (*R*^2^ = 0.2031, *p* < 0.001; Figure 4D), supported by PERMANOVA results (Supplementary Figure 1A).

At the phylum level, *Bacillota* and *Bacillota_A* were dominant across the intestinal tract. *Bacillota* was significantly more abundant in the cecum and colon, while *Bacillota_A* was enriched in the small intestine (*p* < 0.05; Figure 4E and Supplementary Figures 1C,D). *Bacteroidota* also showed higher relative abundance in the large intestine (*p* < 0.05; Supplementary Figure 1E). Genus-level analysis revealed reduced abundances of *Ligilactobacillus*, *Limosilactobacillus*, and *Lactobacillus* in the cecum and colon (*p* < 0.001 and *p* < 0.05, respectively; Supplementary Figures 1F–H).

The potential for BA metabolism, assessed by the richness and Shannon diversity of BA-related genes, varied substantially along the intestinal tract (Supplementary Figures 2A–C). Shannon diversity was highest in the cecum, and colon, whereas the duodenum exhibited the lowest diversity. A similar trend was observed for gene richness, with the ileum harboring the highest number of BA-metabolizing genes and the duodenum again showing the lowest. These findings suggest that BA-transforming potential is regionally specialized, with limited activity in the proximal small intestine and enhanced metabolic capacity in the distal gut.

### 3.5 Alterations in BA-microbiota signature following *Salmonella typhimurium* infection

**Figure 5:**
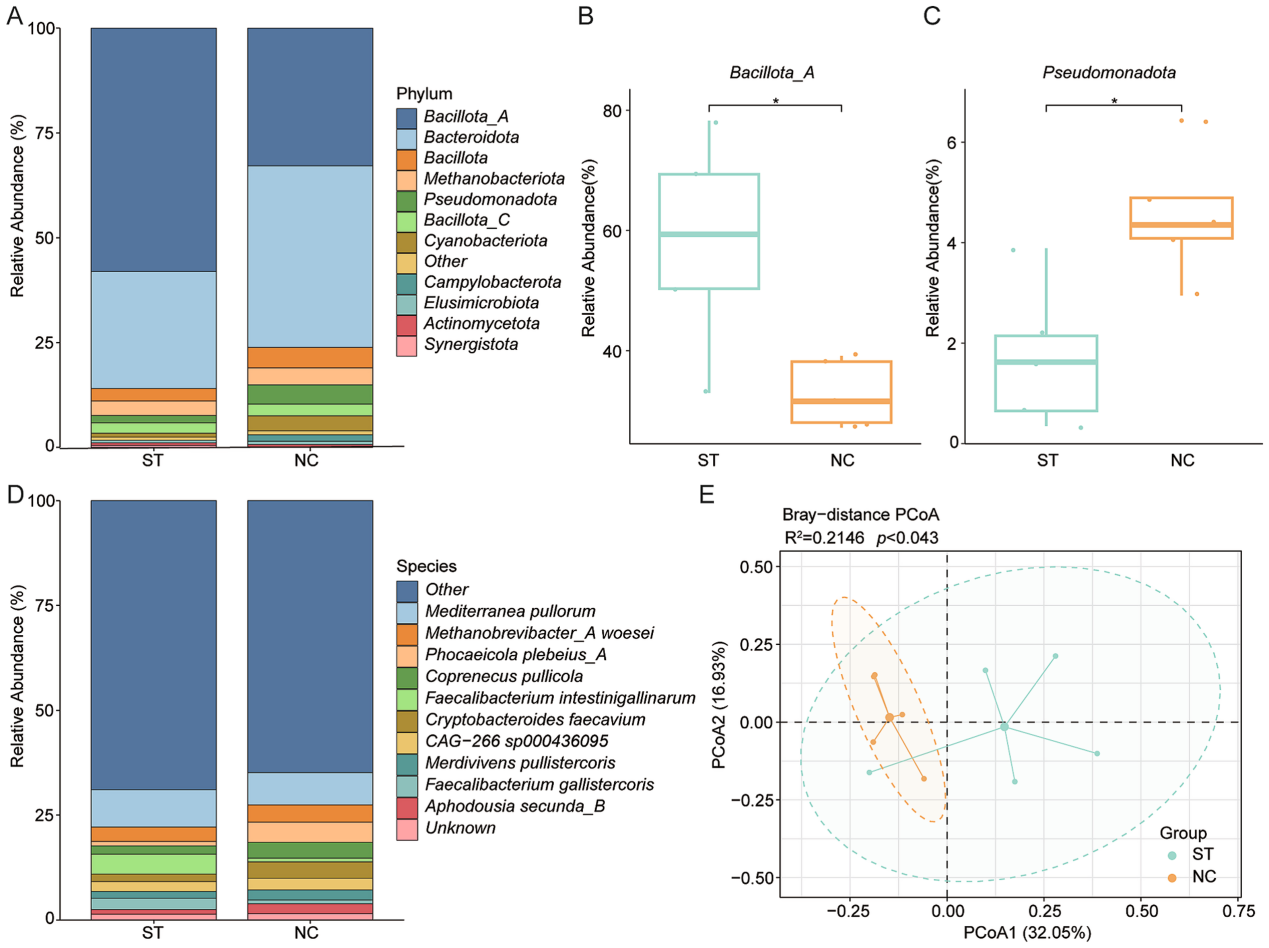
Impact of *Salmonella typhimurium* infection on BA-related gut microbiota. **(A)** Phylum-level community composition in *S. typhimurium*-infected (ST) and control (NC) groups. **(B,C)** Relative abundances of *Bacillota_A* and *Pseudomonadota* in ST versus NC groups. **(D)** Genus-level comparison of microbial community composition between ST and NC groups. **(E)** PCoA based on Bray–Curtis distances illustrating β-diversity between infected and control microbiomes. Statistical significance was determined using Wilcoxon rank-sum test: *p < 0.05.

To investigate the impact of *S. typhimurium* infection on BA-related microbiota, the gut metagenomes of infected chickens were reanalyzed using the curated BA gene dataset. At the phylum level, *Bacillota_A* and *Bacteroidota* remained the most abundant taxa (Figure 5A). Interestingly, *Bacillota_A* was significantly enriched in infected chickens, whereas *Pseudomonadota* was reduced (*p* < 0.05; Figures 5B,C). At the species level, *Mediterranea pullorum* and *Methanobrevibacter_A woesei* were the most prevalent in infected samples (Figure 5D). Conversely, *Phocaeicola plebeius_A* and *Limisoma sp900544305* were significantly reduced (*p* < 0.001; Supplementary Figures 3A,B), while *Faecalibacterium intestinigallinarum* and *Mediterraneibacter excrementipullorum* showed increased abundance (*p* < 0.05; Supplementary Figures 3C,D). PCoA revealed a clear separation between infected and control (CON) groups (*p* = 0.043; Figure 5E), indicating infection-associated shifts in community structure. In addition, the relative abundance of *BSH* genes differed significantly between groups (Supplementary Figure 3E), suggesting that *S. typhimurium* infection alters not only taxonomic composition, but also functional potential related to BA metabolism. Further analysis revealed a decreased prevalence of BA biosynthesis enzyme genes in the infected groups compared with the controls (Supplementary Figure 3F). Alpha diversity analysis (richness indices) showed that the richness of *7α-HSDH* was significantly lower in the infected groups than in the controls (*p* < 0.05, Wilcoxon rank-sum test; Supplementary Figure 3G).

### 3.6 Impact of *Eimeria tenella* infection on BA-related gut microbiota

**Figure 6:**
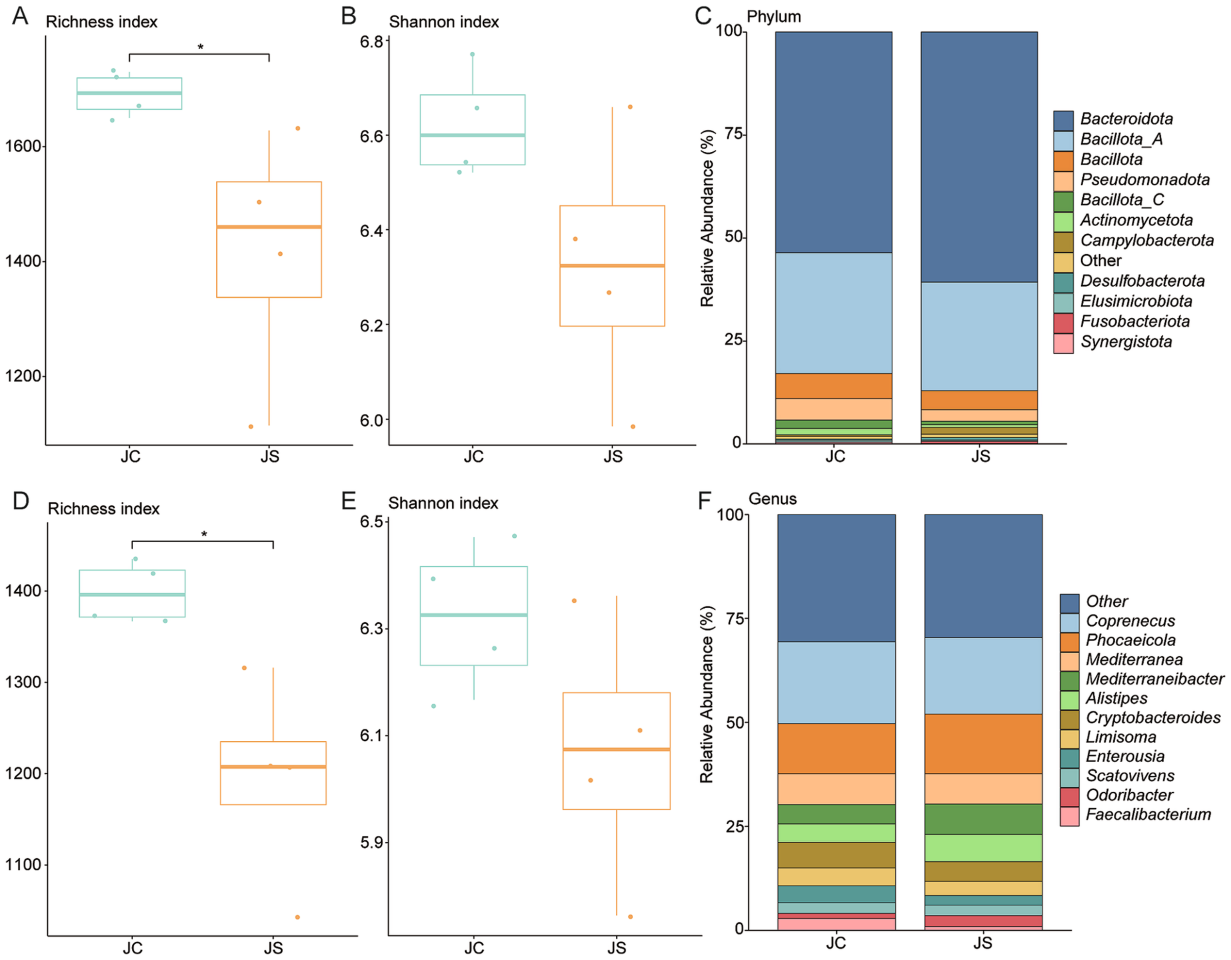
Alterations in BA-metabolizing gut microbiota following *Eimeria tenella* infection. **(A,B)** Boxplots of richness and Shannon diversity indices in control (JC) and infected (JS) groups. **(C)** Phylum-level taxonomic composition of BA-related genomes in JC and JS groups. **(D,E)** Diversity indices for genomes encoding *BSH* genes across groups. **(F)** Genus-level taxonomic composition of *BSH*-encoding genomes in JC and JS groups. JS: *E. tenella*-infected group; JC: control group. Statistical significance was determined using Wilcoxon rank-sum test: *p < 0.05.

To evaluate the impact of *E. tenella* infection on BA-associated gut microbiota, we reanalyzed metagenomic data from a previous study (32) using a curated BA biosynthesis gene set. Following infection, alpha diversity metrics revealed a significant increase in richness but a decrease in Shannon diversity, suggesting reduced community evenness despite higher species count (Figures 6A,B). At the phylum level*, Bacteroidota* was dominant, followed by *Bacillota_A* and *Bacillota* (Figure 6C). Importantly, *Synergistota* was significantly more abundant in the control group (*p* < 0.05; Supplementary Figure 4A). At the species level, *Coprenecus pullicola*, *Mediterranea pullorum*, and *Phocaeicola barnesiae* were most prevalent, while *Scatomorpha stercorigallinarum* was significantly enriched in controls (*p* < 0.05; Supplementary Figures 4B,C). A similar trend was observed in *BSH* gene-carrying genomes: Shannon diversity was lower in the infected group, indicating reduced functional diversity related to bile salt deconjugation (Figures 6D,E). The most abundant *BSH* gene- harboring genera were *Coprenecus*, *Phocaeicola*, and *Mediterranea* (Figure 6F). At the species level, *Lactobacillus crispatus* was also more abundant in the control group (*p* < 0.05; Supplementary Figures 4D,E). Further analysis revealed an increased abundance of *7α-HSDH* in the infected groups compared to the controls (Supplementary Figure 4F).

## 4 Discussion

We performed genome-resolved metagenomic and functional profiling of BA-transforming microbiota in the chicken intestine. We reconstructed 9,990 non-redundant genomes across 23 phyla and 708 genera. Our analyses reveal considerable spatial and taxonomic variations, as well as functional diversity in BA metabolism. The dominance of *Bacillota_A*, *Bacteroidota*, and *Bacillota*, with high representation from *Lachnospiraceae* and *Lactobacillaceae*, supports previous findings (45) and reflects adaptation to the avian gut environment and its nutrient dynamics. These dominant phyla contribute to BA metabolism through complementary functions: *Bacillota*, including *Lachnospiraceae* and *Lactobacillaceae*, specialize in fermentation, short-chain fatty acid production (which has anti-inflammatory effects) (46) and bile salt metabolism, whereas *Bacteroidota* focus on polysaccharide degradation and immune signaling (47). Their synergy is driven by ecological complementarity, not phylogenetic relatedness, and supports efficient fat digestion, immune regulation, and resilience.

To understand the functional potential of the chicken gut microbiome in BA metabolism, we assessed the genomic capacity for BA transformation, focusing on key enzymes involved in bile salt deconjugation and secondary BA synthesis. Functional annotation analysis reveals that 80.17% of chicken gut genomes encoded genes related to BA metabolism, particularly *BSH* genes, which were widely distributed across 12 phyla and present in 4,186 genomes. However, genes involved in downstream transformations, such as *7α-HSDH* and *baiB*, were much less prevalent, with *7α-HSDH* found in only nine phyla and *baiB* limited to three phyla. This uneven gene distribution indicates a critical bottleneck: while BA deconjugation is widespread, full secondary BA biosynthesis is restricted to a small microbial subset.

This genomic distribution aligns with spatial patterns observed along the chicken intestinal tract. Microbial diversity is higher in the cecum and colon than in the small intestine, a pattern consistent with previous studies and attributed to the longer retention times and anaerobic conditions characteristic of the large intestine (48–51). Along the intestinal tract, taxonomic composition shifts markedly: *Bacillota_A* dominates the small intestine, while *Bacillota* and *Bacteroidota* are more abundant in the cecum and colon (52). *BSH*-positive genera such as *Ligilactobacillus* and *Limosilactobacillus* were enriched in the proximal gut. This suggests that deconjugation activity is highest near the point of bile entry, promoting early bile salt modification and enhancing lipid solubilization. In contrast, the distal gut, characterized by more anaerobic conditions, hosts microbial taxa better suited for secondary BA synthesis (53), although their lower genomic abundance may limit the overall production of signaling-active BAs.

The widespread presence of *BSH* genes, particularly in genera such as *Ligilactobacillus* and *Alistipes*, reflects evolutionary adaptation to bile salt pressure in the chicken gut, enabling stable microbial colonization despite the antimicrobial properties of conjugated BAs. Spatially, proximal small intestine microbiota favor deconjugation, while the distal gut supports secondary BA synthesis, which is limited in chickens. These patterns reflect evolutionary, dietary, and physiological adaptations affecting nutrient metabolism, immune regulation, and pathogen resistance. These spatial and functional insights into BA metabolism have important implications for host health because secondary BAs play key roles in regulating lipid metabolism, immune responses, and pathogen resistance. Therefore, understanding the distribution and limitations of BA-transforming capabilities in the gut microbiome may inform nutritional or probiotic strategies aimed at enhancing fat digestion, modulating host metabolism, and promoting gut health. Specifically, rye-based diets reduce conjugated BA concentrations in the chicken small intestine and impair fat digestion through microbial shifts; effects that can be reversed by supplementation with xylanase and *β*-glucanase (54). The enrichment of *BSH*-carrying taxa such as *Ligilactobacillus* and *Limosilactobacillus* in the small intestine aligns with this site of physiological impact, suggesting that microbial deconjugation activity directly influences host lipid metabolism and intestinal absorption. Given the limited prevalence of downstream BA transformation genes, preserving conjugated BAs may be especially critical in avian systems.

This microbial constraint on secondary BA synthesis is underscored by recent findings that supplementation with secondary BAs, such as hyodeoxycholic acid (HDCA), mitigates metabolic stress effects in broilers (55). Chronic corticosterone induces fatty liver and hepatic glucocorticoid receptor downregulation, but dietary HDCA reverses these effects, improving lipid metabolism and stress resilience. To place these findings in a broader context, we compared BA-metabolizing gene repertoires across chickens, humans, and pigs. This analysis revealed distinct host-specific profiles. Chickens had the highest *BSH* gene prevalence, highlighting their dominant role in BA metabolism, but the lowest *baiA* gene abundance, confirming a functional bottleneck in secondary BA synthesis. Complementary studies in pigs show that dietary BA supplementation modifies serum and fecal BA profiles and host metabolism, even without significant microbiome shifts (56). These findings support the concept that exogenous BA supplementation can bypass microbial limitations and enhance metabolic outcomes.

Broiler chicken studies further demonstrate that dietary BA supplementation mitigates heat stress-induced hepatic lipid accumulation by downregulating lipogenic gene expression, reducing liver triglycerides, and maintaining endogenous BA biosynthesis (57). Swine-derived BA supplementation improves growth performance, carcass traits, and intestinal lipase activity (58), while other studies highlight reduced abdominal fat, lower serum triglycerides, favorable lipid metabolism modulation, and enhanced hepatic fatty acid oxidation (12, 59). BA supplementation also alters liver BA composition and gut microbiota differently under low- and high-fat diets, linking microbial shifts to improved lipid metabolism and liver health. These findings not only enhance our understanding of BA metabolism in poultry but also highlight the broader implications for metabolic health, immune function, and disease resistance in livestock systems. By modulating BA profiles, we may be able to influence the gut microbiota’s capacity to regulate nutrient absorption and inflammation, ultimately improving animal health and productivity. Studies reveal dynamic crosstalk between BA metabolism and gut microbiota in conditions such as non-alcoholic fatty liver disease in chickens. Here, diet-induced microbial dysbiosis alters BA profiles and liver health (13). Fasting also modulates BA metabolism through negative feedback in liver and ileum, mediated by host-microbiota metabolic interactions involving metabolites such as L-valine (14). Collectively, these findings underscore the complexity of BA-microbiota-host interactions and the potential of dietary strategies to optimize health and performance in poultry production.

The role of BA metabolism extends beyond microbial composition; enteric infections, *S. typhimurium* or *E. tenella* can disrupt BA metabolism, leading to dysbiosis and impaired nutrient absorption (60). In agreement with previous studies (60, 61), *S. typhimurium* challenge does significantly alter microbial *α* diversity, but induce marked shifts in *β* diversity, indicating significant restructuring of microbial community composition. Interestingly, infected birds exhibited enrichment of *Bacillota_A* and a concurrent depletion of *Pseudomonadota*, indicative of a shift toward *Bacillota* -dominated communities. These taxonomic changes were accompanied by substantial turnover in BA-transforming taxa, including, a reduction in key *BSH*-carrying species such as *Phocaeicola plebeius_A*, alongside increases in *Mediterranea pullorum* and *Faecalibacterium intestinigallinarum*.

Functionally, this dysbiosis corresponded with a loss of *BSH* gene diversity and abundance, suggesting reduced BA deconjugation capacity. Such impairments are likely to affect micelle formation, lipid emulsification, and nutrient absorption. More importantly, altered BA availability may disrupt host BA receptor signaling (e.g., FXR, TGR5), with downstream effects on metabolism, immune function, and inflammation (62, 63). These disruptions to the BA-microbiota axis may compromise mucosal integrity, promote inflammation, and exacerbate susceptibility to infection-associated pathology. Interestingly, dietary BA supplementation has been shown to counteract *S. typhimurium*-induced dysbiosis, restoring microbial balance, enhancing goblet cell abundance and mucin *MUC2* gene expression, and reducing pathogen colonization (60). This highlights the therapeutic potential of targeted BA interventions in mitigating pathogen-induced gut dysfunction.

Similarly, *E. tenella* infection induces alterations in microbial composition and function, consistent with previous findings (26, 28, 30, 64). While α-diversity increased due to higher species richness, there was a marked decline in evenness and *BSH* gene richness, indicative of ecological imbalance and dominance of select taxa. Although *Bacteroidota* remained the dominant phylum, substantial losses were observed in beneficial taxa such as *Caccocola* and *Lactobacillus crispatus*, known contributors to BA metabolism, immune modulation, and epithelial homeostasis. These losses likely impair the generation of free and secondary BAs, thereby weakening their antimicrobial, anti-inflammatory, and barrier-supportive functions (7, 8). Consistent with this, previous studies have also reported reductions in *Faecalibacterium*, *Ruminococcaceae UCG-013*, *Romboutsia*, and *Shuttleworthia*, together with increases in opportunistic pathogens such as *Enterococcus* and *Streptococcus* (27). These compositional shifts suggest a breakdown of the cecal microbial ecosystem, potentially heightening vulnerability to secondary infections and mucosal damage.

The contraction in functional redundancy for BA metabolism during *E. tenella* infection underscores the importance of microbial-derived BAs in maintaining intestinal homeostasis. Dysregulation of BA receptor signaling further implicates these metabolic disruptions in shaping host inflammatory responses (62, 63). Moreover, the parasite’s dependence on the microbiota introduces a paradox: while *E. tenella* development appears to require a functionally intact microbial community, infection itself disrupts that very ecosystem. This is supported by evidence of impaired parasite development in germ-free chickens (65), where absence of microbiota, and hence BA metabolism, limits *E. tenella* replication. Interestingly, even in germ-free birds with reduced parasite burden, *BSH* activity remained disrupted, suggesting that both infection-induced dysbiosis and microbiota absence converge on shared metabolic vulnerabilities.

Collectively, these findings underscore the sensitivity of the BA-microbiota axis to enteric infections and its central role in host–pathogen interactions. The maintenance of microbial functional capacity, particularly *BSH* gene diversity, appears crucial for preserving gut homeostasis and host resilience. Interventions that restore or sustain BA-transforming taxa, such as probiotics or targeted nutritional strategies, may help break the cycle of infection-induced dysbiosis, reduce disease severity, and support intestinal health. Finally, while our data demonstrate strong associations between infection, microbiota shifts, and functional outcomes, it remains essential to disentangle causality. Future studies employing targeted metabolomics and *in vitro* validation of microbial enzymatic activities are warranted to clarify the mechanistic links between pathogen challenge, BA metabolism, and host physiology.

## 5 Conclusion

This study highlights a fundamental constraint in the chicken gut microbiome: the widespread ability to deconjugate bile salts contrasts with a limited capacity for complete secondary BA synthesis. The marked spatial variation in microbial communities, along with infection-driven dysbiosis following exposure to *S. typhimurium* and *E. tenella* highlights the finely tuned nature of BA metabolism to the local intestinal environment and its vulnerability to disruption. These findings highlight the importance of region-specific microbial functions in maintaining metabolic homeostasis and reveal the sensitivity of the BA-microbiota axis to perturbations caused by infection, which may impact lipid digestion, immune responses, and host-pathogen interactions. To enhance poultry health and productivity, future research should focus on optimizing BA metabolic pathways, including through nutritional interventions and microbiome modulation. Since our study was based on known genes from KEGG pathways, complementary efforts should aim to identify novel BA-related enzymes through *de novo* gene discovery, which may uncover previously unrecognized mechanisms shaping host–microbiota interactions.

## Data Availability

The original contributions presented in the study are included in the article/Supplementary material, further inquiries can be directed to the corresponding authors.

## Supplementary material

SUPPLEMENTARY FIGURE 1 Regional variation in microbial composition along the chicken intestine. **(A)** PERMANOVA results showing the overall effect size of intestinal site on microbial composition. Pairwise comparisons between intestinal segments are shown in the bottom-left panel. *p*-values were calculated using the adonis function with 1,000 permutations in R. **(B)** Stacked bar plots displaying phylum-level composition of the gut microbiota across five intestinal regions. **(C–E)** Boxplots showing the relative abundance of *Bacillota*, *Bacillota_A*, and *Bacteroidota* by region. **(F–H)** Boxplots showing the relative abundance of *Ligilactobacillus*, *Limosilactobacillus*, and *Lactobacillus* across intestinal sites. Statistical significance was assessed using the Wilcoxon rank-sum test. Asterisks indicate significance levels: **p* < 0.05; ***p* < 0.01; ****p* < 0.001. DOU – duodenum; JEJ – jejunum; ILE – ileum; CEC – cecum; COL – colon.

SUPPLEMENTARY FIGURE 2 Regional variation of BA genes along the intestinal tract. **(A–C)** Boxplots showing the relative abundance of key BA transformation genes (BSH, *7α-HSDH*, *baiB*) across different intestinal regions. Statistical significance was assessed using the Wilcoxon rank-sum test. Asterisks indicate significance levels: **p* < 0.05; ***p* < 0.01; ****p* < 0.001. DOU–duodenum; JEJ–jejunum; ILE–ileum; CEC–cecum; COL–colon.

SUPPLEMENTARY FIGURE 3 Microbial shifts in response to *Salmonella typhimurium* infection. **(A–D)** Boxplots showing the relative abundance of *Phocaeicola plebeius_A*, *Limisoma sp900544305*, *Faecalibacterium intestinigallinarum*, and *Mediterraneibacter excrementipullorum* in the *S. typhimurium*-infected (ST) and control (NC) groups. **(E)** Principal Coordinates Analysis (PCoA) based on Bray–Curtis distance, illustrating α-diversity differences in BSH gene profiles between ST and NC groups. **(F)** Relative abundance of bile acid metabolism-related enzyme genes in the ST and NC groups. **(G)** Boxplots of richness index of *7α-HSDH* across the ST and NC groups. Significance was determined using the Wilcoxon rank-sum test. Asterisks indicate significance level: **p* < 0.05.

SUPPLEMENTARY FIGURE 4 Alterations in BA-related gut microbiota in response to *Eimeria tenella* infection. **(A)** Boxplot showing the relative abundance of *Synergistota* in infected (JS) and control (JC) groups. **(B)** Stacked bar plot depicting species-level microbial community composition in JC and JS groups. **(C)** Boxplots showing the relative abundance of *Scatomorpha stercorigallinarum* in JC and JS groups. **(D)** Stacked bar plot summarizing species-level community composition differences between JC and JS groups. **(E)** Boxplot showing the relative abundance of *Lactobacillus crispatus* in JC and JS groups. Significance was evaluated using the Wilcoxon rank-sum test. Asterisks indicate significance level: *, *p* < 0.05. **(F)** Relative abundance of three bile acid metabolism-related enzyme genes in the JC and JS groups. JS: *E. tenella*-infected group; JC: control group.

SUPPLEMENTARY TABLE 1 Metadata for all intestinal samples and associated sequencing datasets used in this study.

SUPPLEMENTARY TABLE 2 Summary statistics of the 9,990 high-quality microbial genomes, including completeness, contamination, genome size, and taxonomic classification.

SUPPLEMENTARY TABLE 3 List of 8,009 genomes involved in bile acid transformation pathways, including gene annotations for BSH, *7α-HSDH*, and *baiB*.

SUPPLEMENTARY TABLE 4 Comparative analysis of bile acid metabolism-related genes, including data for 2,294 human MAGs **(a)** and for 1,411 pig MAGs **(b)**.
