# Supplementary figures and images for: Genome-centric investigation of bile acid-metabolizing microbiota in chickens and their association with Eimeria tenella and Salmonella typhimurium infections
Source: Front Vet Sci. 2025 Oct 17;12:1669620. doi: 10.3389/fvets.2025.1669620 (PMC12575232; doi:10.3389/fvets.2025.1669620)

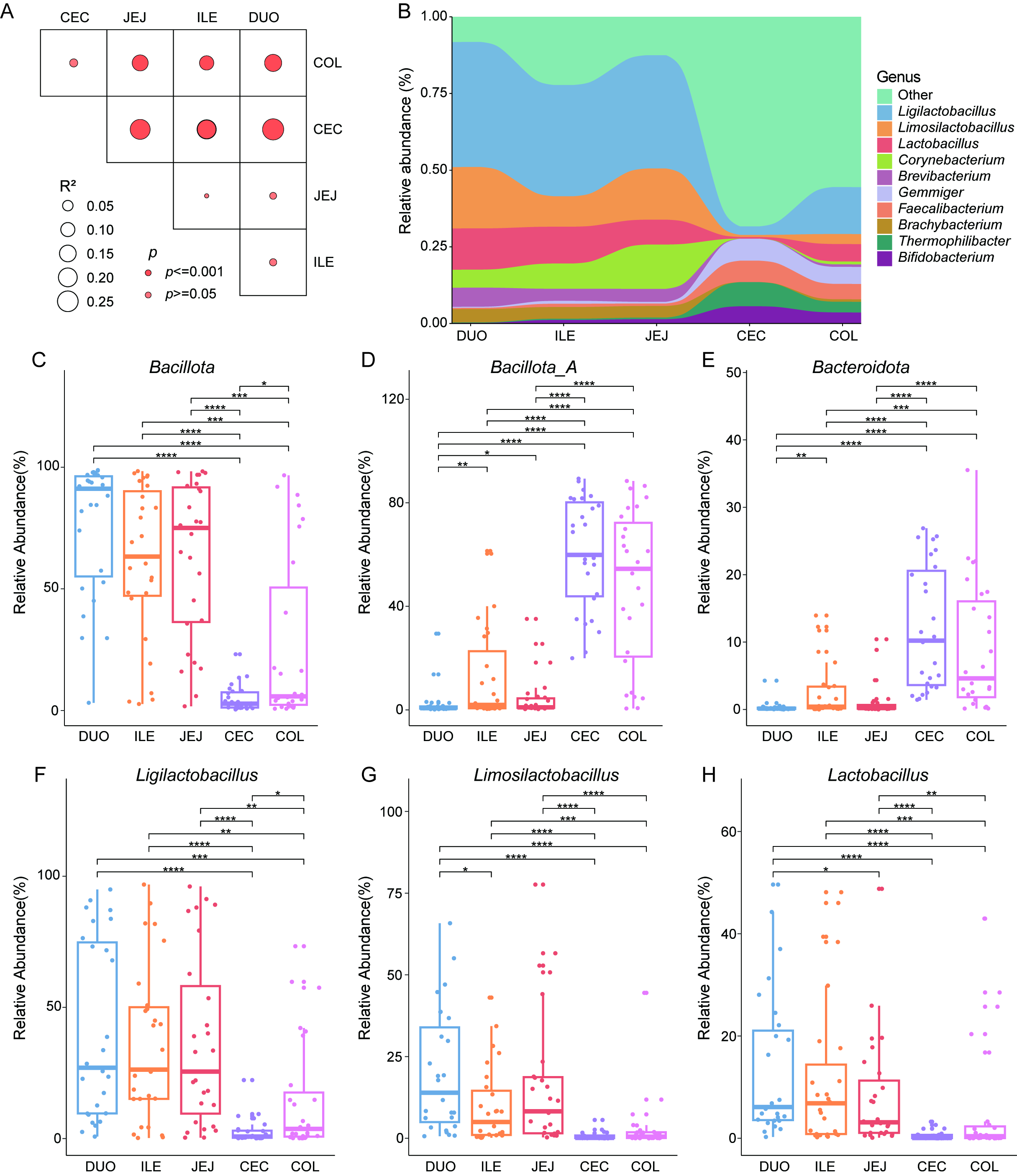

Supplement: SUPPLEMENTARY FIGURE 1 — Regional variation in microbial composition along the chicken intestine. (A) PERMANOVA results showing the overall effect size of intestinal site on microbial composition. Pairwise comparisons between intestinal segments are shown in the bottom-left panel. p-values were calculated using the adonis function with 1,000 permutations in R. (B) Stacked bar plots displaying phylum-level composition of the gut microbiota across five intestinal regions. (C–E) Boxplots showing the relative abundance of Bacillota, Bacillota_A, and Bacteroidota by region. (F–H) Boxplots showing the relative abundance of Ligilactobacillus, Limosilactobacillus, and Lactobacillus across intestinal sites. Statistical significance was assessed using the Wilcoxon rank-sum test. Asterisks indicate significance levels: *p < 0.05; **p < 0.01; ***p < 0.001. DOU – duodenum; JEJ – jejunum; ILE – ileum; CEC – cecum; COL – colon. [file Image_1.TIF]

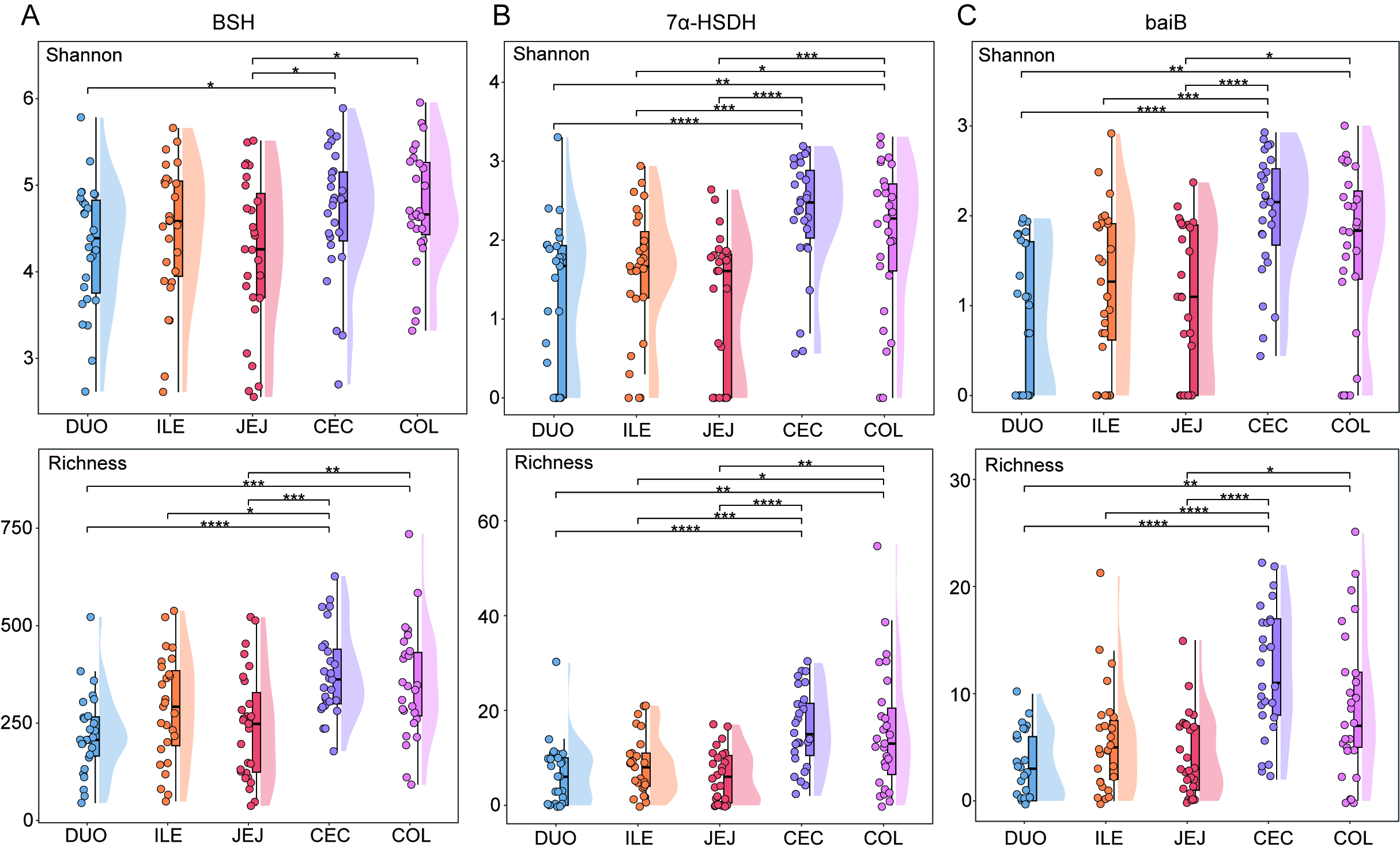

Supplement: SUPPLEMENTARY FIGURE 2 — Regional variation of BA genes along the intestinal tract. (A–C) Boxplots showing the relative abundance of key BA transformation genes (BSH, 7α-HSDH, baiB) across different intestinal regions. Statistical significance was assessed using the Wilcoxon rank-sum test. Asterisks indicate significance levels: *p < 0.05; **p < 0.01; ***p < 0.001. DOU–duodenum; JEJ–jejunum; ILE–ileum; CEC–cecum; COL–colon. [file Image_2.TIF]

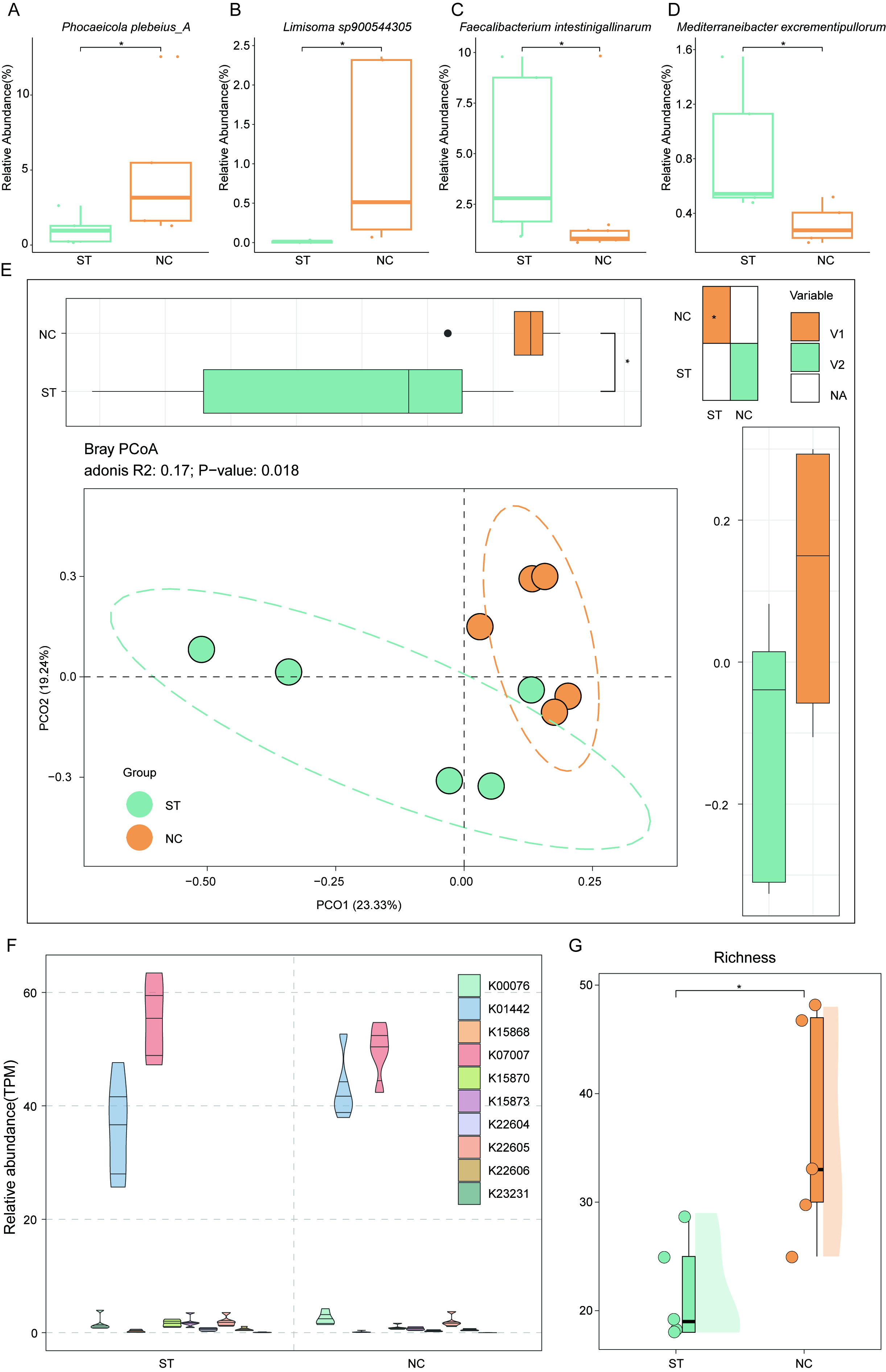

Supplement: SUPPLEMENTARY FIGURE 3 — Microbial shifts in response to Salmonella typhimurium infection. (A–D) Boxplots showing the relative abundance of Phocaeicola plebeius_A, Limisoma sp900544305, Faecalibacterium intestinigallinarum, and Mediterraneibacter excrementipullorum in the S. typhimurium-infected (ST) and control (NC) groups. (E) Principal Coordinates Analysis (PCoA) based on Bray–Curtis distance, illustrating α-diversity differences in BSH gene profiles between ST and NC groups. (F) Relative abundance of bile acid metabolism-related enzyme genes in the ST and NC groups. (G) Boxplots of richness index of 7α-HSDH across the ST and NC groups. Significance was determined using the Wilcoxon rank-sum test. Asterisks indicate significance level: *p < 0.05. [file Image_3.TIF]

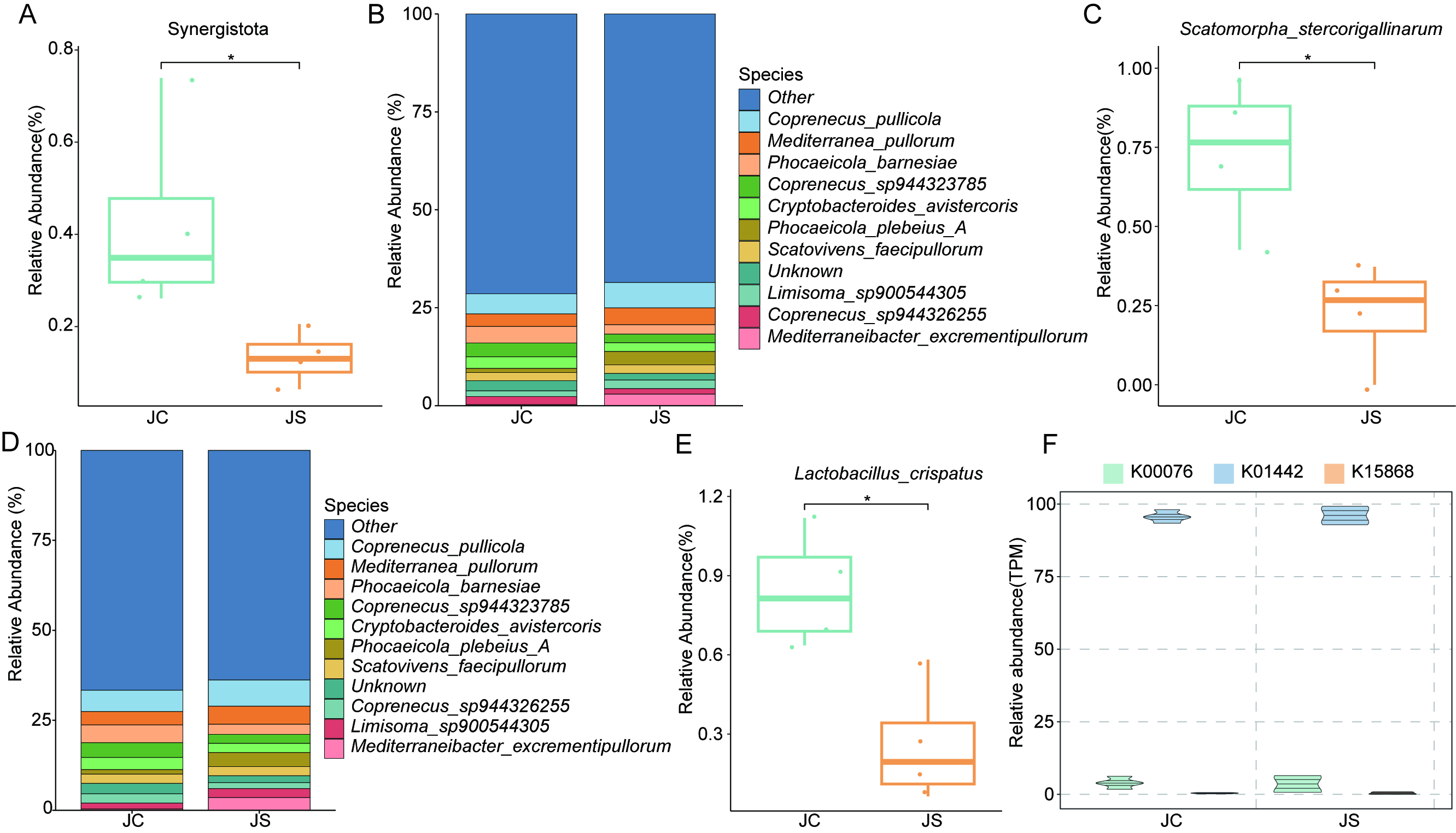

Supplement: SUPPLEMENTARY FIGURE 4 — Alterations in BA-related gut microbiota in response to Eimeria tenella infection. (A) Boxplot showing the relative abundance of Synergistota in infected (JS) and control (JC) groups. (B) Stacked bar plot depicting species-level microbial community composition in JC and JS groups. (C) Boxplots showing the relative abundance of Scatomorpha stercorigallinarum in JC and JS groups. (D) Stacked bar plot summarizing species-level community composition differences between JC and JS groups. (E) Boxplot showing the relative abundance of Lactobacillus crispatus in JC and JS groups. Significance was evaluated using the Wilcoxon rank-sum test. Asterisks indicate significance level: *, p < 0.05. (F) Relative abundance of three bile acid metabolism-related enzyme genes in the JC and JS groups. JS: E. tenella-infected group; JC: control group. [file Image_4.TIF]
